# Supplementary material for: A Mycobacterium tuberculosis surface protein recruits ubiquitin to trigger host xenophagy
Source: Nat Commun. 2019 Apr 29;10:1973. doi: 10.1038/s41467-019-09955-8 (PMC6488588; doi:10.1038/s41467-019-09955-8)
Supplement: Supplementary file 1 — Supplementary Information [file 41467_2019_9955_MOESM1_ESM.docx]

**Supplementary Information**

**A *Mycobacterium tuberculosis* surface protein recruits ubiquitin to trigger host xenophagy**

Chai *et al.*

**
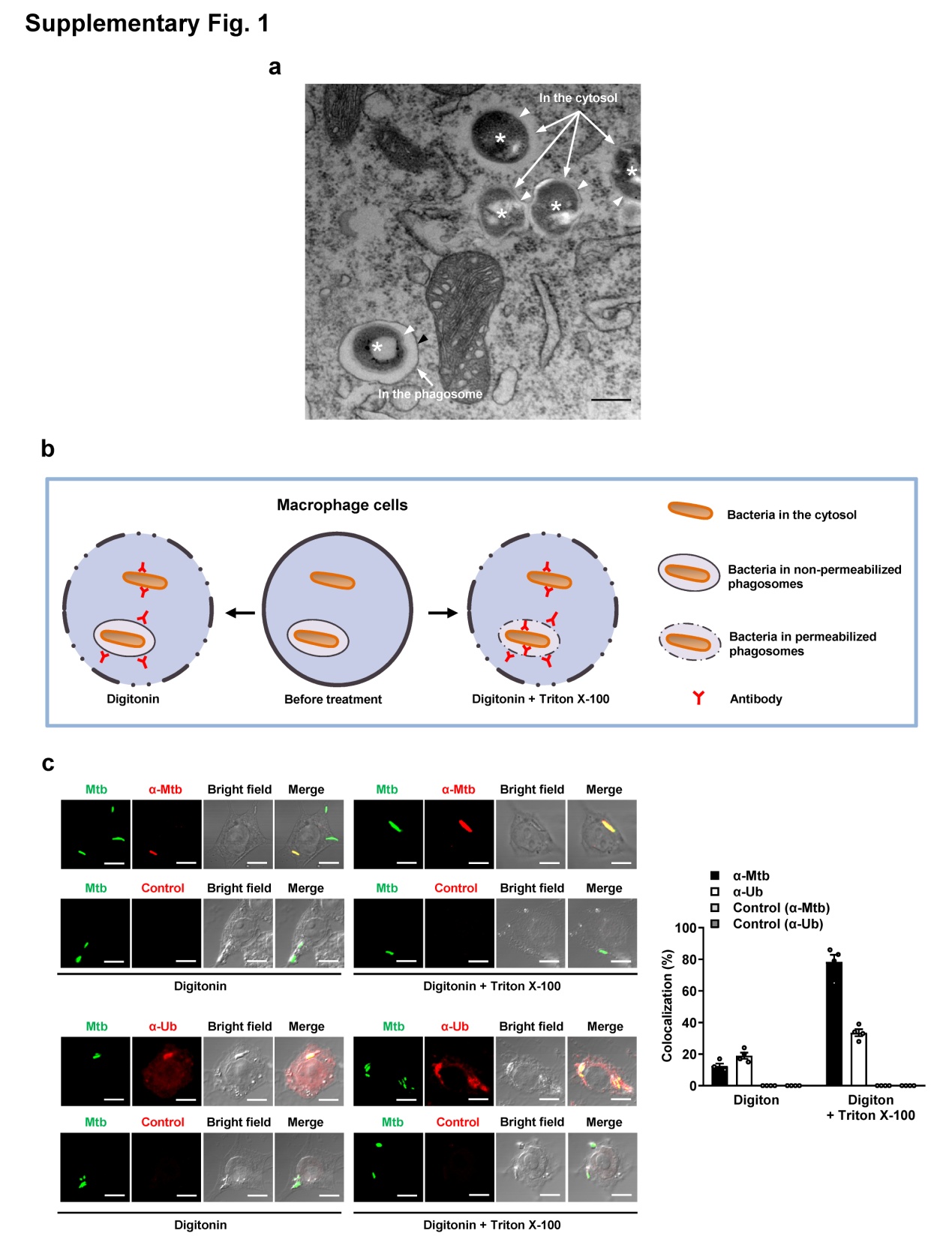
**

**Supplementary Fig. 1** Ubiquitin binds to mycobacterial surface in macrophages. (**a**) Electron microscopy analysis for localization of *Mycobacterium tuberculosis* (Mtb) in bone marrow-derived murine macrophages (BMDMs). BMDMs were infected with Mtb H37Rv for 4 h. Asterisks represent the mycobacterial cells. White arrowhead, the bacterial wall; black arrowhead, the phagosomal membrane. Scale bars, 500 nm. (**b**) Schematic diagram of different treatments for permeabilization of macrophage cells and the antibody accessibility. (**c**) Confocal microscopy analysis for colocalization of ubiquitin (Ub) with Mtb in infected BMDMs. BMDMs were infected with Mtb H37Rv for 12 h and were then immunostained using anti-Mtb or anti-Ub antibody (red). Bacteria (green) were prestained with Alexa Fluor 488 succinimidyl ester before infection. Staining controls for anti-Ub and anti-Mtb were done with mouse IgG1 or without primary antibody, respectively. Cells were permeabilized by using digitonin only or digitonin plus Triton X-100 for different membrane permeabilization as indicated in **b**. Scale bars, 5 µm. Right, percent colocalization of Ub with Mtb in BMDMs. A total of 100 bacterial cells were counted. Results are representatives from three independent experiments (mean ± s.e.m. of *n* = 4 in **c**). The source data used in **c** are provided in Source Data.**
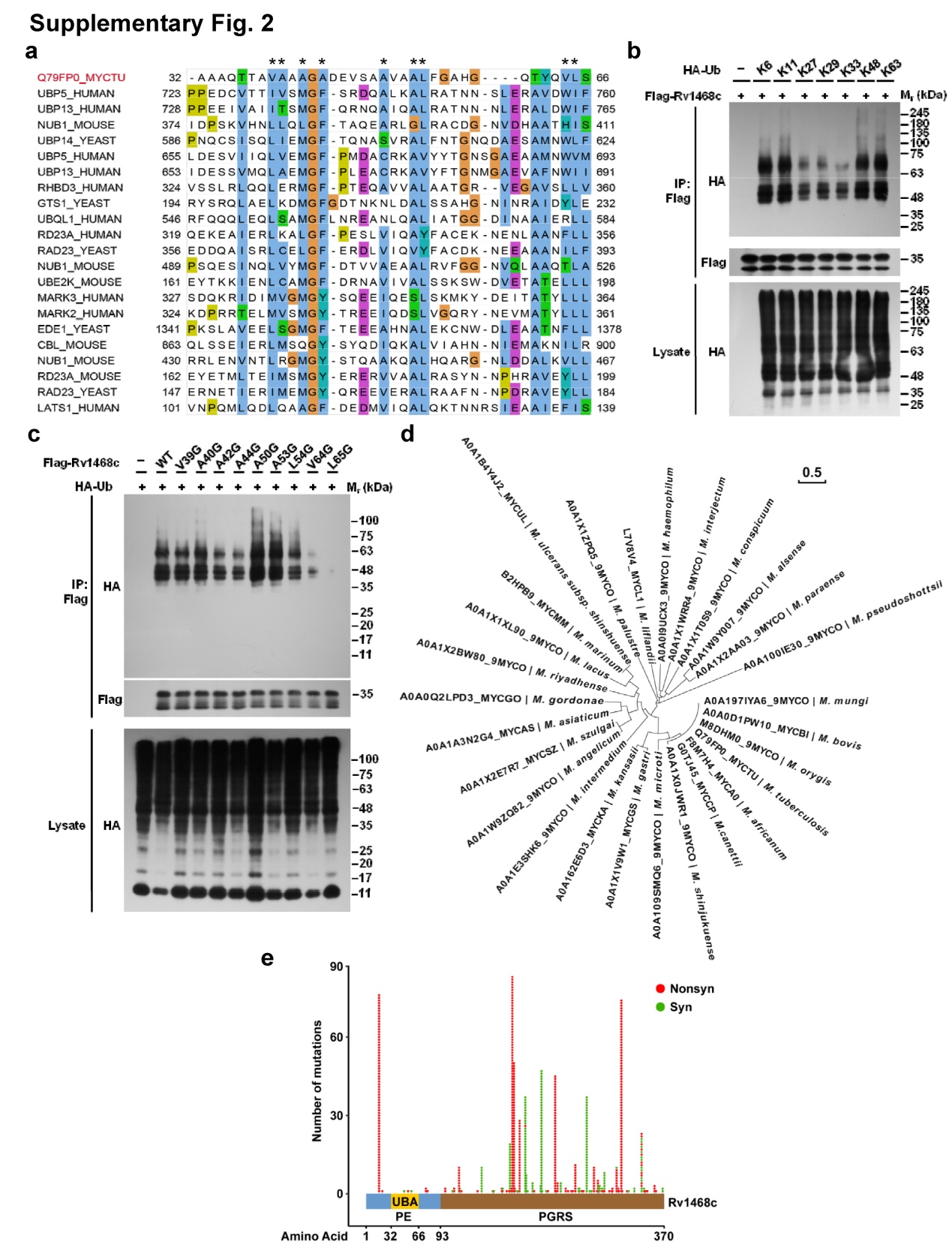
**

**Supplementary Fig. 2** Mtb Rv1468c interacts with poly-Ub chains via UBA domain. (**a**) Sequence comparison of eukaryotic UBAs (blank) and Mtb Rv1468c UBA (red). The analysis was performed with Jalview 2.10.2b2 using PFAM database (Blue, hydrophobic; magenta, negative charge; green, polar; orange, glycines; yellow, prolines). Asterisks mark the mutated sites of Rv1468c used in **c**. (**b**) Coimmunoprecipitation of Mtb Rv1468c (+) with or without (–) each of seven linkage types of Ub chains from the lysates of HEK293T cells cotransfected with Flag-Rv1468c and HA-tagged K6 only, K11 only, K27 only, K29 only, K33 only, K48 only or K63 only Ub. (**c**) Immunoblot analysis of proteins immunoprecipitated (IP) with anti-Flag M2 Affinity Gel from lysates of HEK293T cells transfected with empty vector (–) or hemagglutinin (HA)-tagged Ub (+) and Flag-tagged wild-type (WT) Rv1468c or its mutants (V39G, A40G, A42G, A44G, A50G, A53G, L54G, V64G or L65G) as indicated in **a**. (**d**) Phylogenetic analysis of Rv1468c homologues in mycobacterial species acquired by blasting of Mtb Rv1468c against UniProtKB_Bacteria database. (**e**) Schematic representation of Mtb Rv1468c with mutation sites. The numbers of synonymous (green) or nonsynonymous (red) mutations in Rv1468c were identified in clinical Mtb isolates from GMTV database and plotted by using R version 3.4.4. See also Supplementary Data 1. Results are representatives from at least three independent experiments for **b** and **c**.

**
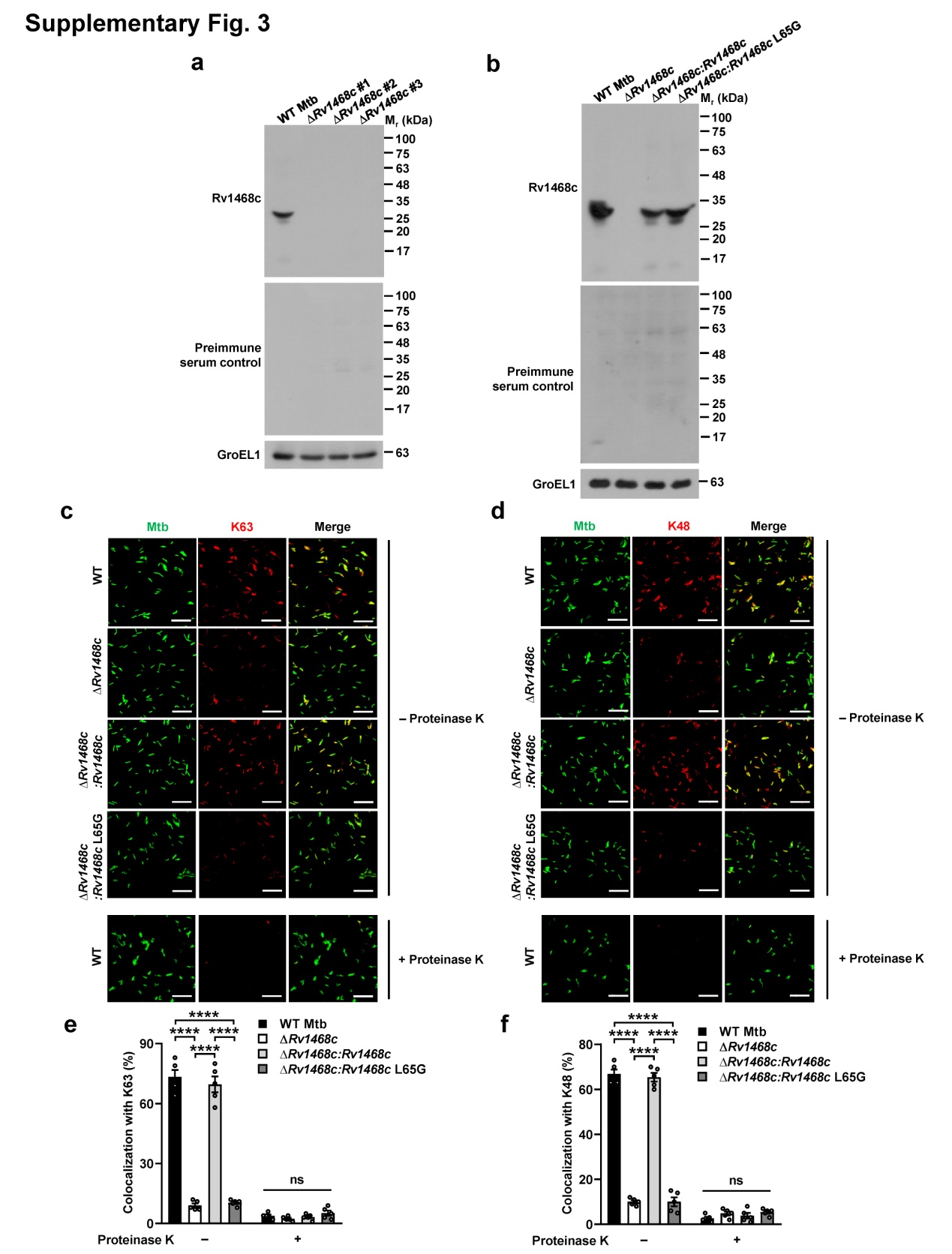
**

**Supplementary Fig. 3** Rv1468c is required for ubiquitin binding to Mtb *in vitro*. **a**, **b** Immunoblot analysis for the expression of Rv1468c and GroEL1 (loading control throughout) in WT strains, *Rv1468c* mutant strains (∆*Rv1468c* #1, #2 and #3) (**a**) and the complemented strains (∆*Rv1468c:Rv1468c* and ∆*Rv1468c:Rv1468c* L65G) (**b**) of Mtb H37Rv. Each strain was grown in Middlebrook 7H9 broth at 37 ℃ to the mid-log phase and solubilized in SDS sample buffer. Approximately 50 μg of whole cell lysate of each strain was subjected to SDS-PAGE and blotted with anti-Rv1468c or anti-GroEL1 antibody. **c**, **d** Confocal microscopy analysis for colocalization of Mtb with Ub chains *in vitro*. Each of the indicated Mtb strains was pretreated with proteinase K (+) or not (–) and was then incubated with K63^2-7^ (**c**) or K48^2-7^ (**d**) poly-Ub chains at 4 ℃ for 4 h, and was then immunostained using anti-Ub antibody (red) followed by staining with Alexa Fluor 488 succinimidyl ester (green). Scale bars, 10 μm. **e**, **f** Percent colocalization of the indicated Mtb strains with K63^2-7^ (**e**) or K48^2-7^ (**f**) poly-Ub chains treated as in **c** and **d**. A total of 100 bacterial cells were counted. *P* > 0.05, not significant (ns); *********P* < 0.0001 (two-way ANOVA). Results are representatives from at least three independent experiments. The source data used in **e** and **f** are provided in Source Data.

**
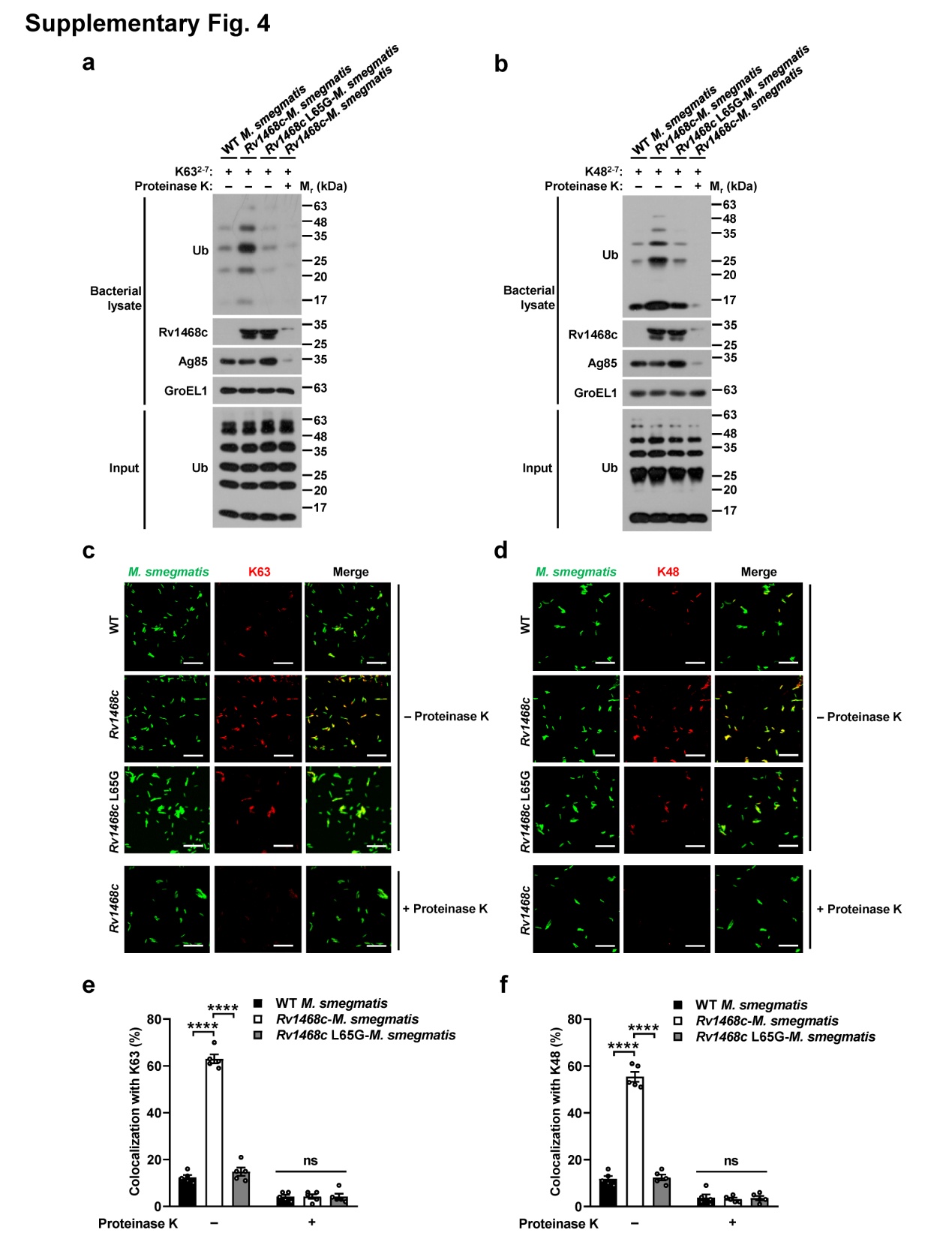
**

**Supplementary Fig. 4** Expression of *Rv1468c* in *M. smegmatis* increases ubiquitin binding to mycobacteria. **a**, **b** Immunoblot analysis of whole bacterial lysate of *M. smegmatis* cells pretreated with (+) or without (–) proteinase K followed by incubation with K63^2-7^ (**a**) or K48^2-7^ (**b**) poly-Ub chains at 4 ℃ for 4 h. **c**, **d** Confocal microscopy analysis for colocalization of *M. smegmatis* with Ub chains. The indicated *M. smegmatis* strains pretreated with (+) or without (–) proteinase K were incubated with K63^2-7^ (**c**) or K48^2-7^ (**d**) poly-Ub chains as in **a** and **b**, and were then immunostained using anti-Ub antibody (red) followed by staining with Alexa Fluor 488 succinimidyl ester (green). Scale bars, 10 μm. (**e**, **f**) Percent colocalizations of the indicated *M. smegmatis* strains with K63^2-7^ (**e**) or K48^2-7^ (**f**) poly-Ub chains treated as in **c** and **d**. A total of 100 bacterial cells were counted. *P* > 0.05, not significant (ns); *********P* < 0.0001 (two-way ANOVA). Results are representatives from at least three independent experiments. The source data used in **e** and **f** are provided in Source Data.


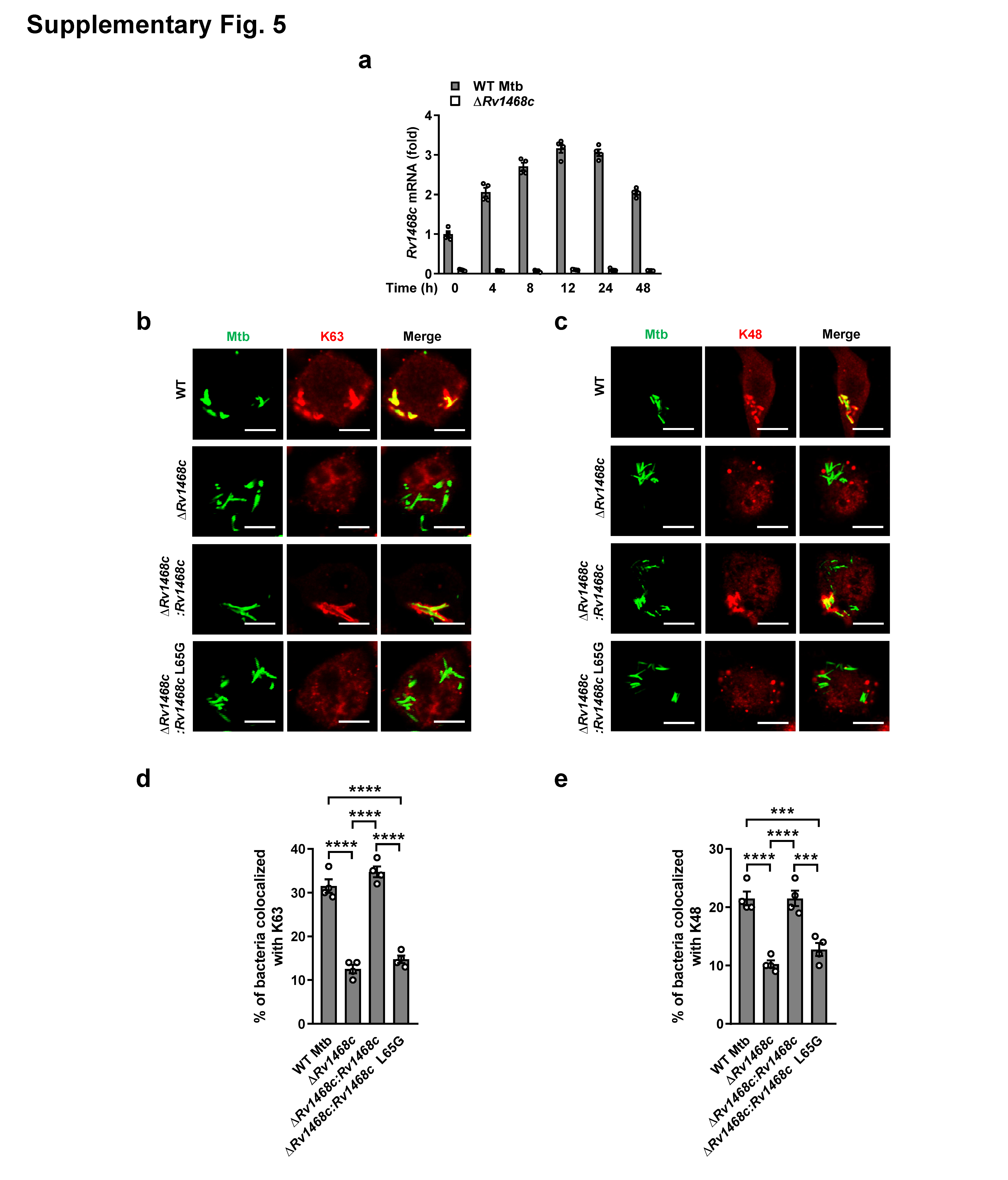


**Supplementary Fig. 5** Mtb Rv1468c is required for colocalization of ubiquitin with Mtb in macrophages. (**a**) Quantitative PCR analysis of *Rv1468c* mRNA in WT Mtb or Mtb ∆*Rv1468c* after infection of BMDMs for 0–48 h. (**b**, **c**) Confocal microscopy analysis for colocalization of Mtb with K63 (**b**) or K48 (**c**) Ub in BMDMs. BMDMs were infected with the indicated Mtb strains at MOI = 5 for 24 h and were then immunostained using anti-K63 or K48 Ub antibody (red). Bacteria (green) were prestained with Alexa Fluor 488 succinimidyl ester before infection. Scale bars, 10 µm. (**d**, **e**) Percent colocalizations of the indicated bacterial strains with K63 (**d**) or K48 (**e**) Ub in macrophages infected as in **b** and **c**. A total of 100 cells were counted. ********P* < 0.001; *********P* < 0.0001 (one-way ANOVA). Results are representatives from at least three independent experiments (mean ± s.e.m. of *n* = 4 in **a**, **d** and **e**). The source data used in **a**, **d** and **e** are provided in Source Data.


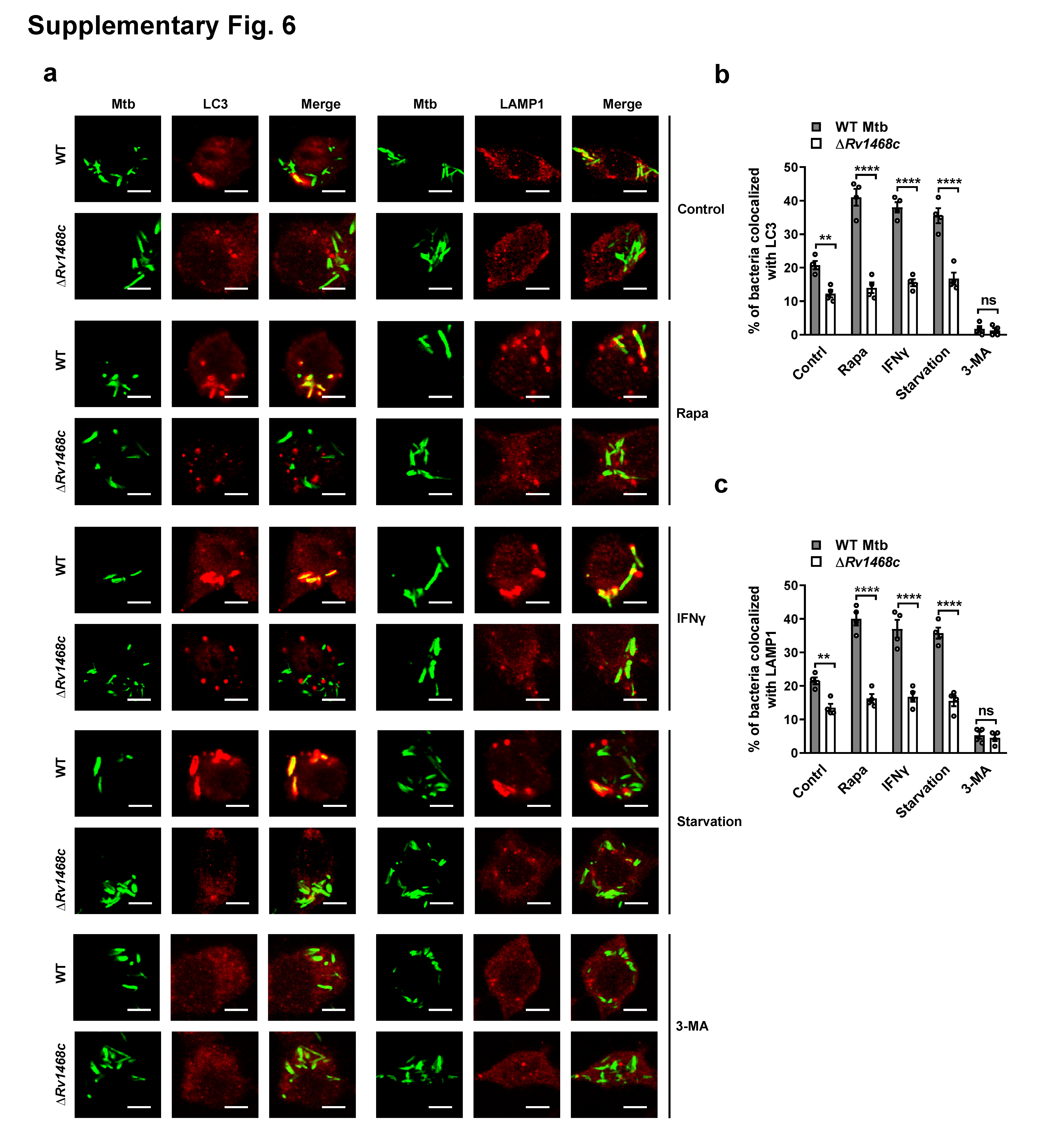


**Supplementary Fig. 6** Deletion of *Rv1468c* reduced colocalization of bacteria with LC3 and LAMP1 in macrophages. (**a**) Confocal microscopy analysis for colocalizations of Mtb with LC3 (left) or LAMP1 (right) in BMDMs. BMDMs were infected with each of the indicated Mtb strains at MOI = 5 for 12 h, and were then immunostained using anti-LC3 or anti-LAMP1 antibody (red). Bacteria (green) were prestained with Alexa Fluor 488 succinimidyl ester before infection. Scale bars, 5 µm. For autophagic induction or inhibition, cells were treated with 50 μM rapamycin (Rapa), 500 U/ml IFNγ, or amino acid and serum starvation for 4 h before infection, or treated with 5 mM 3-methyladenine (3-MA) during the infection. (**b**, **c**) Percent colocalizations of Mtb with LC3 (**b**) or LAMP1 (**c**) in BMDMs treated as in **a**. A total of 100 bacterial cells were counted. *P* > 0.05, not significant (ns); *******P* < 0.01; *********P* < 0.0001 (one-way ANOVA). Results are representatives from three independent experiments (mean ± s.e.m. of *n* = 4 in **b** and **c**). The source data used in **b** and **c** are provided in Source Data.**
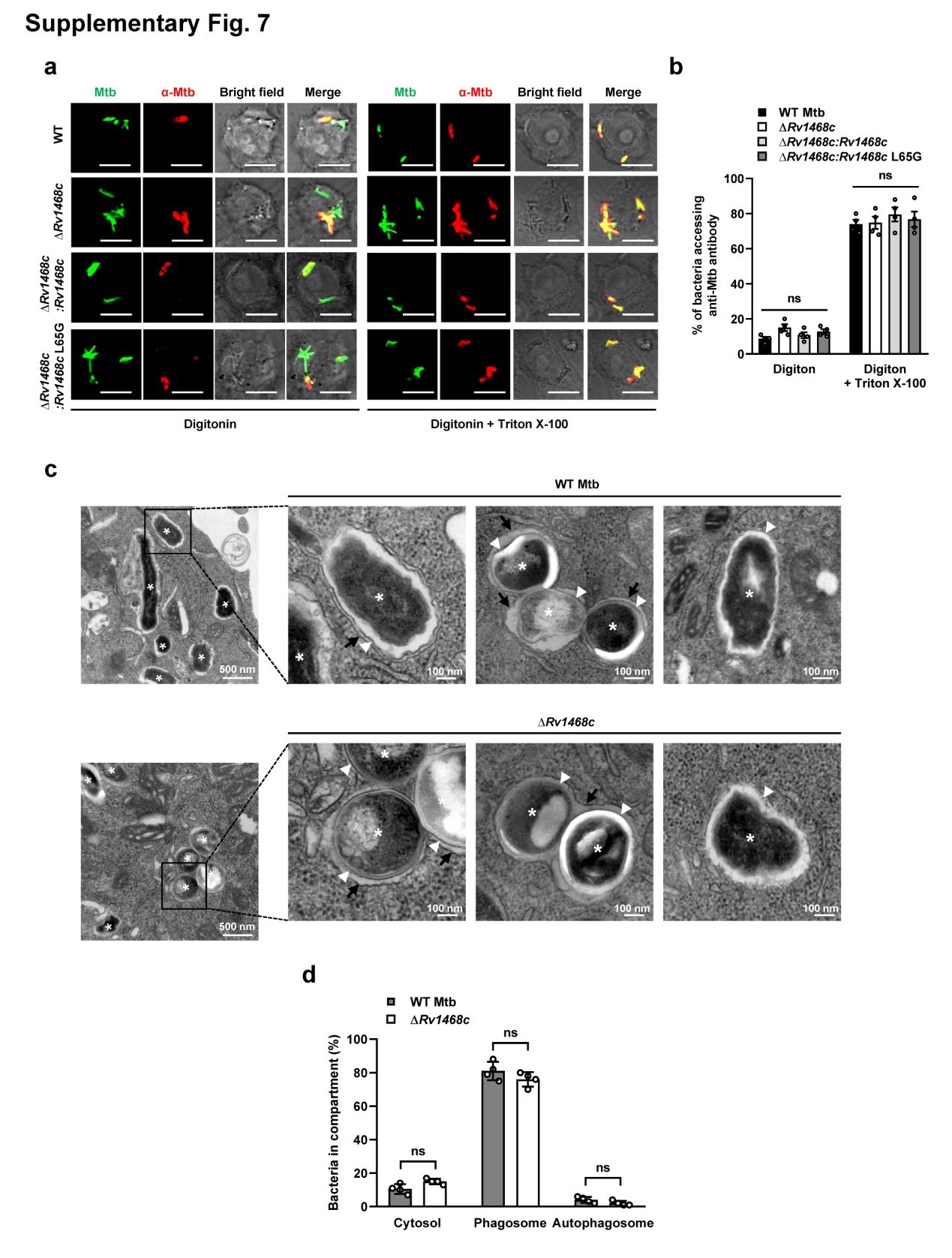
**

**Supplementary Fig. 7** Deletion or mutation of *Rv1468c* does not affect the accessibility of Mtb to the macrophage cytosol. (**a**) Confocal microscopy analysis for the accessibility of anti-Mtb antibody to Mtb in macrophages with different treatments for permeabilization. BMDMs were infected with Mtb H37Rv at MOI = 5 for 4 h, and were then immunostained using anti-Mtb antibody (red). Bacteria (green) were prestained with Alexa Fluor 488 succinimidyl ester before infection. Cells were permeabilized by using digitonin only or digitonin plus Triton X-100 for different membrane permeabilization. (**b**) Quantification of the indicated Mtb strains accessing anti-Mtb antibody in BMDMs treated as in **a**. A total of 100 bacterial cells were counted. *P* > 0.05, not significant (ns; one-way ANOVA). (**c**) Electron microscopy analysis of Mtb subcellular localizations within BMDMs. BMDMs were infected with WT Mtb or Mtb ∆*Rv1468c* as in **a** and were then processed for electron microscopy. Arrows indicate the phagosomal membranes, and arrowheads indicate the bacterial walls. Asterisks represent the mycobacterial cells. (**d**) Quantification of Mtb subcellular localizations within BMDMs treated as in **c**. A total of 100 bacterial cells were counted. *P* > 0.05, not significant (ns; one-way ANOVA). Results are representatives from at least three independent experiments (mean ± s.e.m. of *n* = 4 in **b** and **d**). The source data used in **b** and **d** are provided in Source Data.


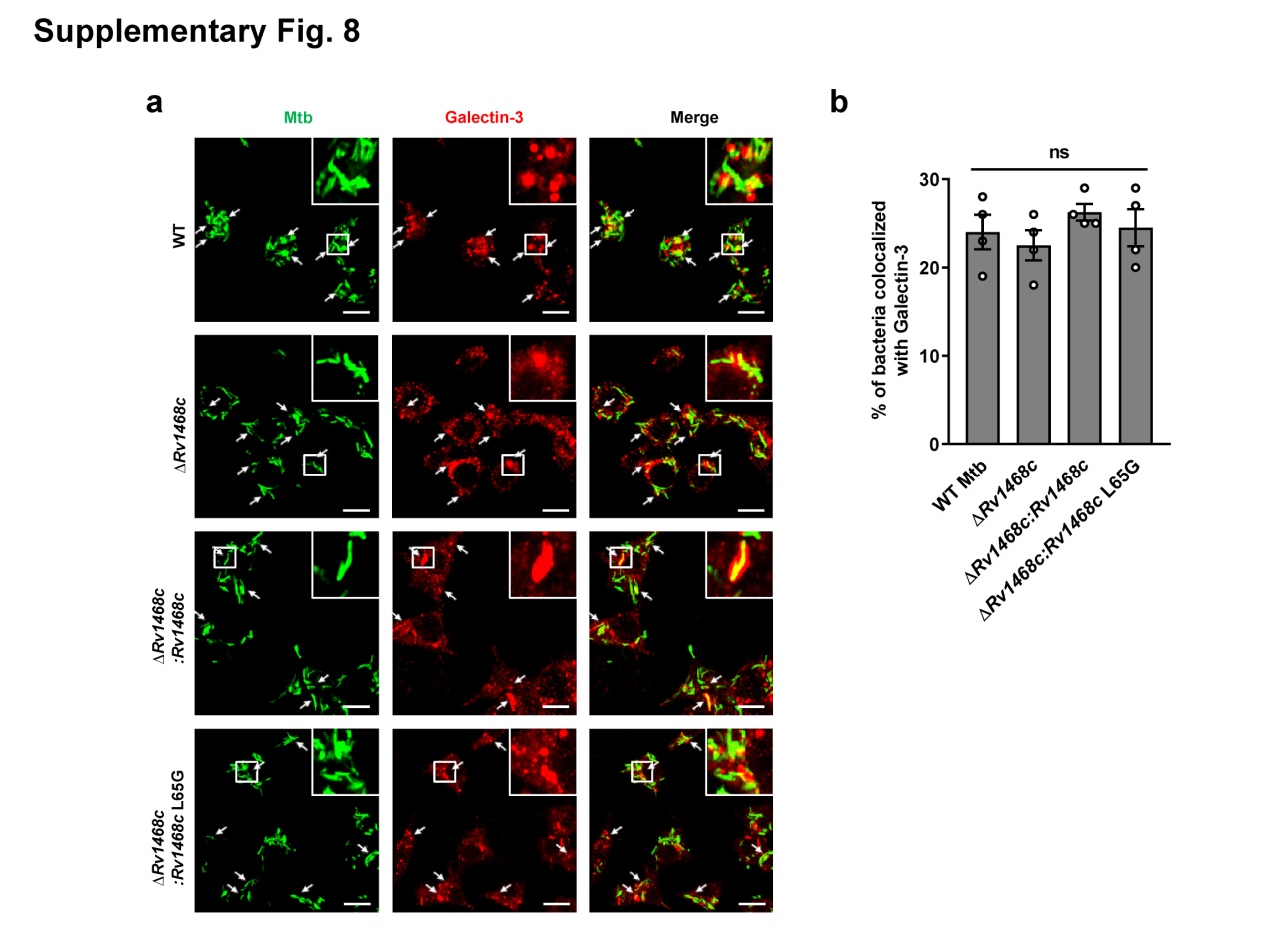


**Supplementary Fig. 8** Deletion or mutation of *Rv1468c* does not affect the colocalization of mycobacteria with Galectin-3 in macrophages. (**a**) Confocal microscopy analysis for colocalization of Mtb with Galectin-3 in BMDMs. BMDMs were infected with the indicated Mtb strains at MOI = 5 for 24 h and were then immunostained using anti-Galectin-3 antibody (red). Bacteria (green) were prestained with Alexa Fluor 488 succinimidyl ester before infection. Arrows indicate the colocalization of bacteria with Galectin-3. Inserts (enlarged views) show representative mycobacteria colocalized with Galectin-3. Scale bars, 20 µm. (**b**) Percent colocalizations of Mtb with Galectin-3 in BMDMs treated as in **a**. A total of 100 bacterial cells were counted. *P* > 0.05, not significant (ns; one-way ANOVA). Results are representatives from three independent experiments (mean ± s.e.m. of *n* = 4 in **b**). The source data used in **b** are provided in Source Data.

**
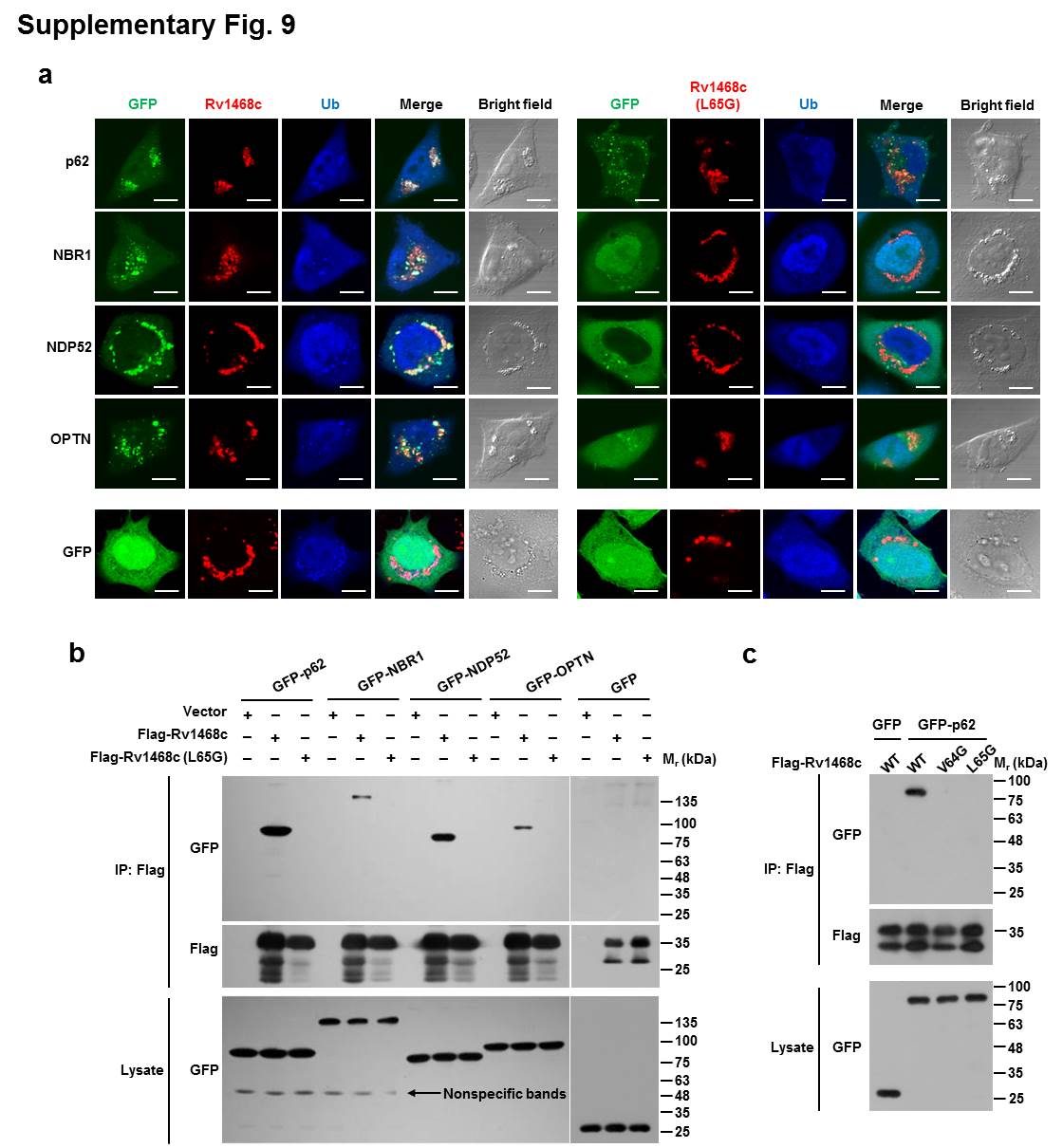
**

**Supplementary Fig. 9** Mtb Rv1468c interacts with autophagy receptors including p62, NBR1, NDP52 and OPTN. (**a**) Confocal microscopy analysis for colocalizations of WT Rv1468c or Rv1468c (L65G) with p62, NBR1, NDP52 and OPTN in HeLa cells. Cells were cotransfected with vectors encoding GFP only, or GFP-tagged p62, NBR1, NDP52, or OPTN (green), DsRed2-tagged Rv1468c or Rv1468c (L65G) (red) and HA-tagged ubiquitin (blue). Scale bars, 5 μm. (**b**) Immunoblot analysis of proteins immunoprecipitated with anti-Flag M2 Affinity Gel from lysates of HEK2937T cells transfected with (+) or without (–) empty vector (control) or vector encoding Flag-tagged Rv1468c or Rv1468c (L65G) and vector encoding GFP, or GFP-tagged p62, NBR1, NDP52 or OPTN. (**c**) Immunoblot analysis of proteins immunoprecipitated as in **b** from lysates of HEK2937T cells transfected with vector encoding Flag-tagged WT Rv1468c, Rv1468c (V64G) or Rv1468c (L65G) and vector encoding GFP or GFP-tagged p62. Results are representatives from at least three independent experiments.


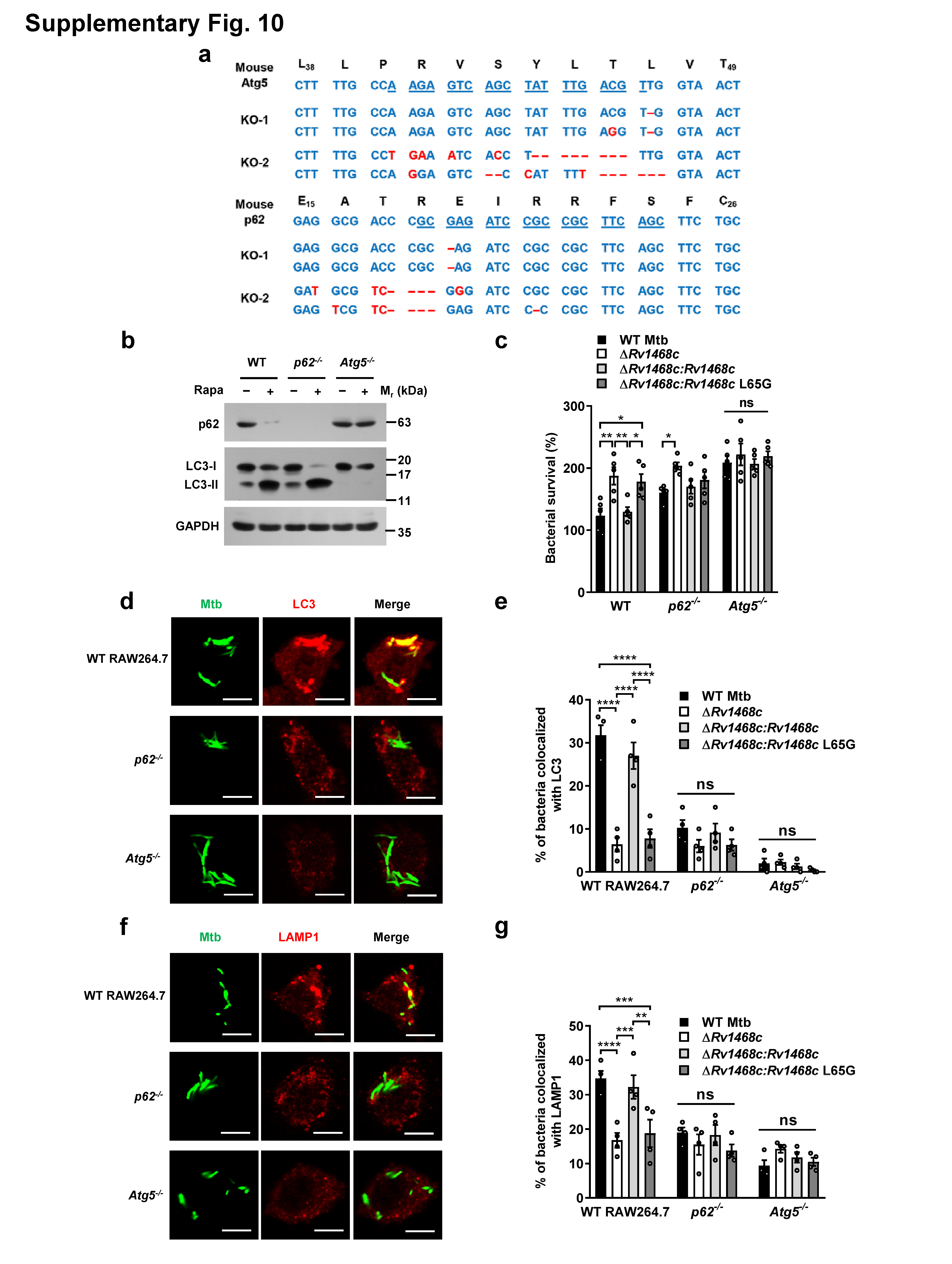


**Supplementary Fig. 10** Deletion of *p62* or *Atg5* impairs Rv1468c-dependent autophagic clearance of mycobacteria. (**a**) Generation of *Atg5^-/-^* or *p62^-/-^* RAW264.7 cells by CRISPR-Cas9-mediated targeting. Shown is the schematic of sequence mutations of the cell clones used in the study. Underlined nucleotides are designated targeting sequence. (**b**) Immunoblot analysis of LC3 and p62 from lysates of WT, *p62^-/-^*, or *Atg5^-/-^* RAW264.7 cells used in **c**–**g** with (+) or without (–) treatment of 50 μM rapamycin (Rapa) for 4 h. (**c**) Survival of Mtb in macrophages. WT, *p62^-/-^* or *Atg5^-/-^* RAW264.7 cells were infected with each of the indicated Mtb strains at MOI = 1 for 24 h. *P* > 0.05, not significant (ns); ******P* < 0.05; *******P* < 0.01 (two-way ANOVA). (**d**, **f**) Confocal microscopy of WT, *p62^-/-^* or *Atg5^-/-^* RAW264.7 cells infected with WT Mtb for 24 h and immunostained using anti-LC3 (**d**) or anti-LAMP1 (**f**) antibody (red). Bacteria (green) were prestained with Alexa Fluor 488 succinimidyl ester before infection. Scale bars, 5 µm. (**e**, **g**) Percent colocalizations of indicated Mtb strains with LC3 (**e**) or LAMP1 (**g**) in macrophages infected as in **d** and **f**. A total of 200 bacterial cells were counted. *P* > 0.05, not significant (ns); *******P* < 0.01; ********P* < 0.001; *********P* < 0.0001 (two-way ANOVA). Results are representatives from three independent experiments (mean ± s.e.m. of *n* = 5 in **c**; *n* = 4 in **e** and **g**). The source data used in **c**, **e** and **g** are provided in Source Data.


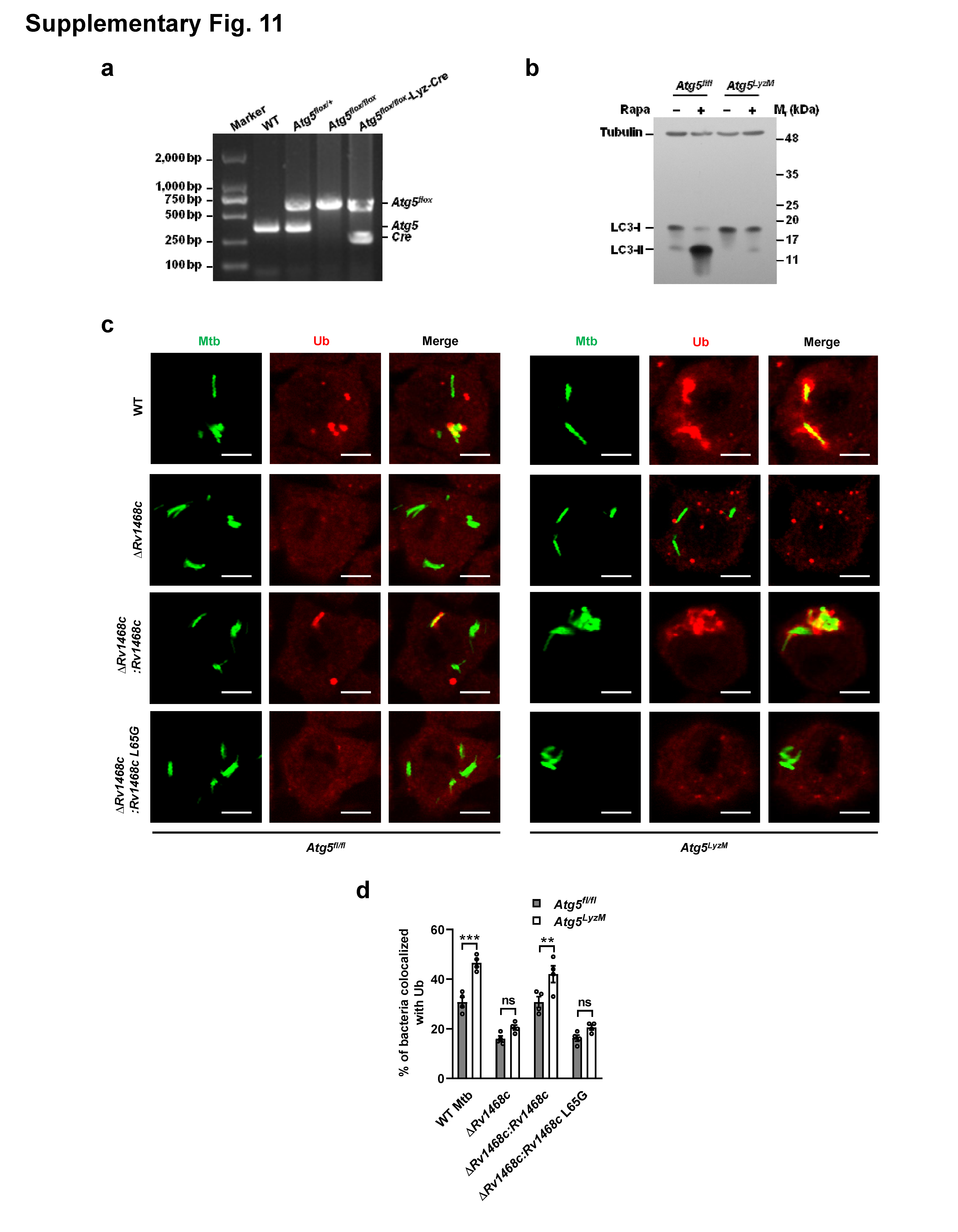


**Supplementary Fig. 11** Rv1468c enhanced Ub accumulation around Mtb in macrophages. (**a**) PCR analysis of BMDMs from WT, *Atg5^flox/+^*, *Atg5^flox/flox^* or *Atg5^flox/flox^*-Lyz-*Cre* C57BL/6 mice. The predicted sizes of PCR products are 700 bp (*Atg5^flox^*), 350 bp (*Atg5*), and 272 bp (*Cre*). (**b**) Immunoblot analysis of LC3 from lysates of BMDMs obtained from *Atg5^flox/flox^* (*Atg5^fl/fl^*) or *Atg5^flox/flox^*-Lyz-*Cre* (*Atg5^LyzM^*) mice treated with (+) or without (–) 50 μM Rapa for 4 h. (**c**) Confocal microscopy analysis for colocalization of Mtb with Ub in BMDMs derived from *Atg5^fl/fl^* or *Atg5^LyzM^* mice. BMDMs were infected with the indicated Mtb strains at MOI = 5 for 24 h and were then immunostained using anti-Ub antibody (red). Bacteria (green) were prestained with Alexa Fluor 488 succinimidyl ester before infection. Scale bars, 5 µm. (**d**) Percent colocalizations of each of indicated Mtb strains with Ub in macrophages infected as in **c**. A total of 100 bacterial cells were counted. *P* > 0.05, not significant (ns); *******P* < 0.01; ********P* < 0.001 (two-way ANOVA). Results are representatives from three independent experiments (mean ± s.e.m. of *n* = 4 in **d**). The source data used in **d** are provided in Source Data.


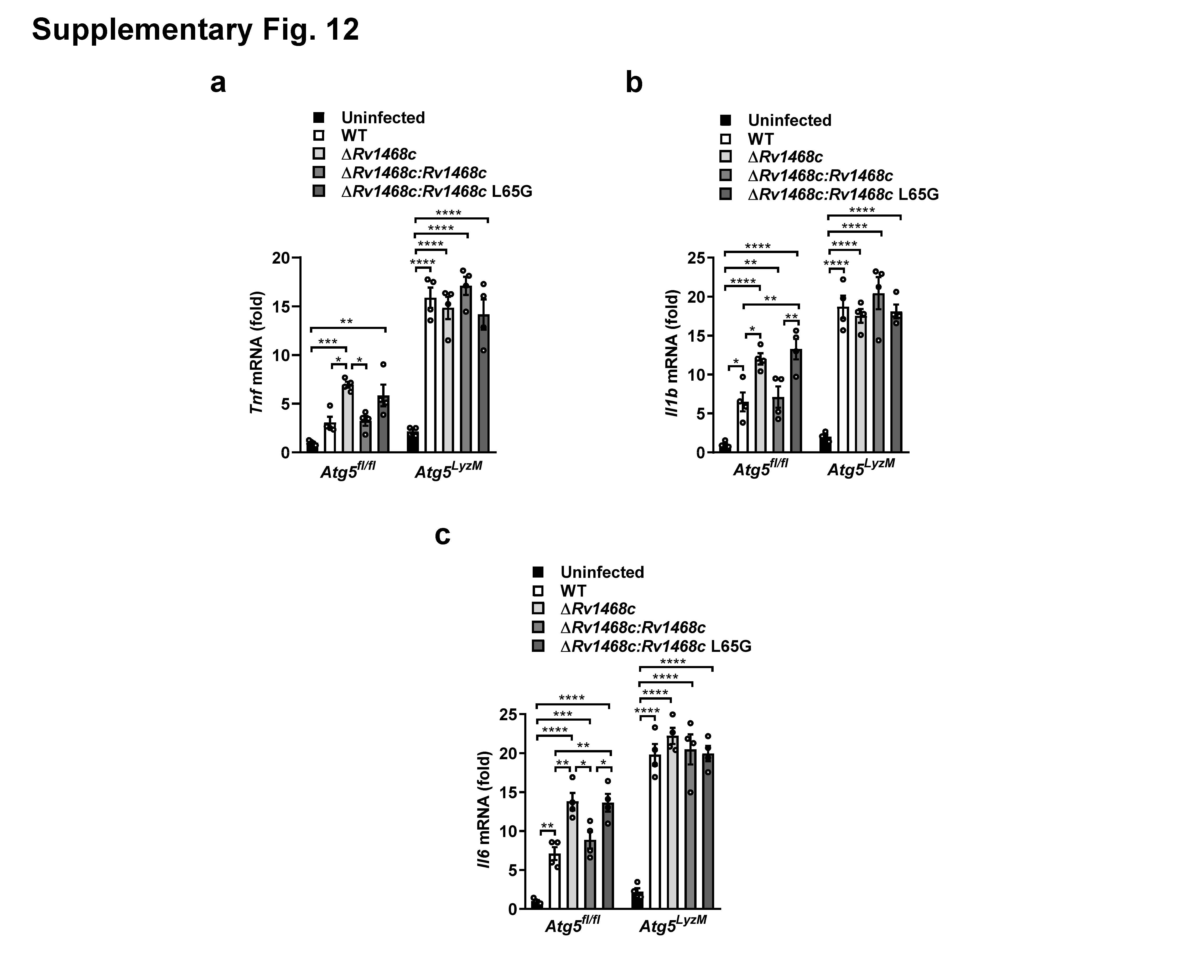


**Supplementary Fig. 12** Cytokines in the lungs of *Atg5^fl/fl^* and *Atg5^LyzM^* mice infected with different Mtb strains. (**a**–**c)** Quantitative PCR analysis of *Tnf* mRNA (**a**), *Il1b* mRNA (**b**) and *Il6* mRNA (**c**) in lungs from *Atg5^fl/fl^* mice or *Atg5^LyzM^* mice intratracheally infected with 1.0 × 10^5^ CFU of the indicated Mtb strains for 3 weeks. ******P* < 0.05; *******P* < 0.01; ********P* < 0.001; *********P* < 0.0001 (two-way ANOVA). Results are representatives from at least three independent experiments (mean ± s.e.m. of *n* = 4 in **a**–**c**). The source data used in **a**–**c** are provided in Source Data.


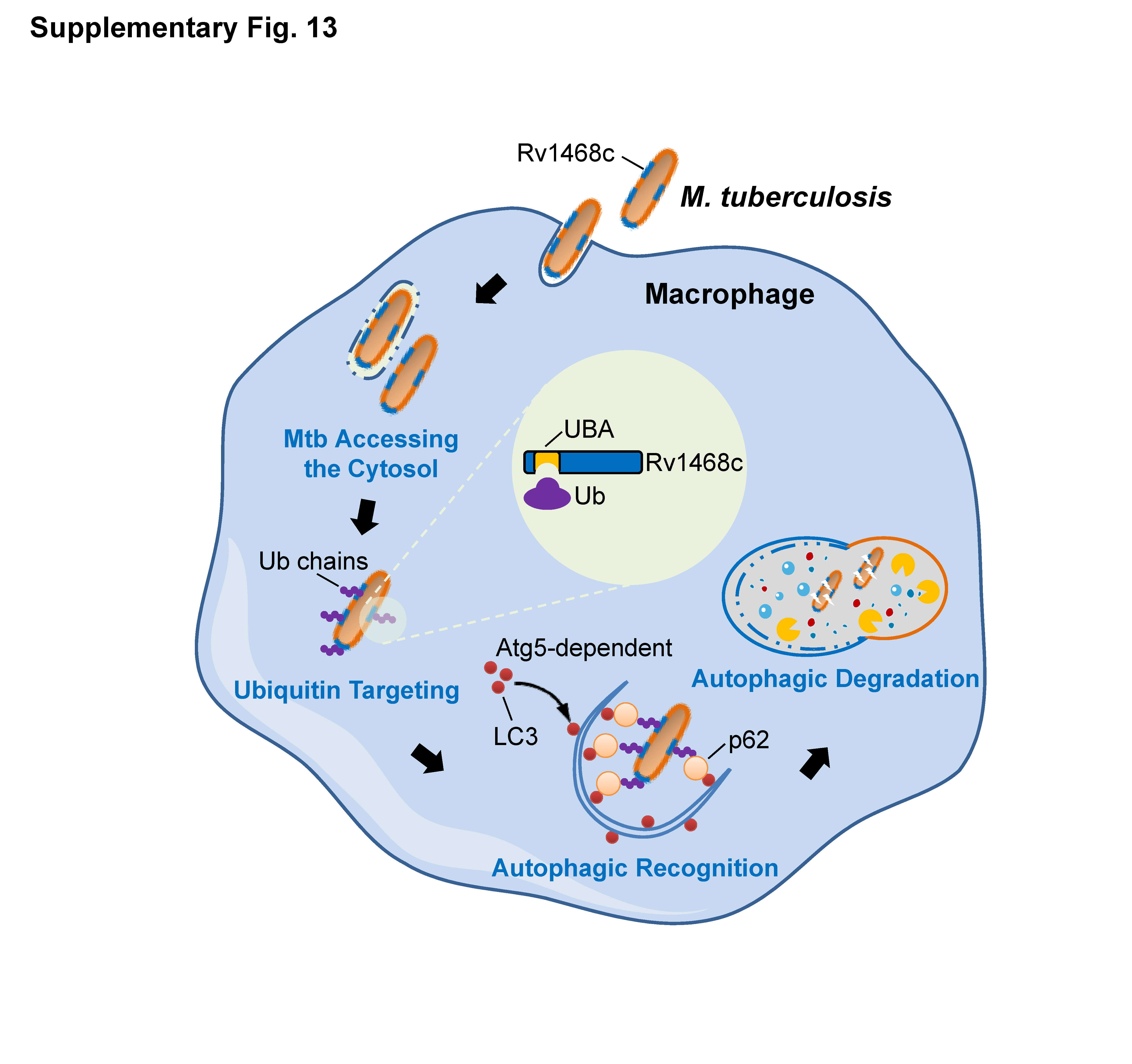


**Supplementary Fig. 13** Schematic model showing the mechanism of ubiquitin-Rv1468c interaction-mediated anti-mycobacterial xenophagy.

**Supplementary Methods**

**Sequence analysis**

For sequence alignment, the sequences in UBA family (PF00627) were downloaded from PFAM database (http://pfam.xfam.org/)^1^. The UBA domain (32–66 amino acids) of Mtb H37Rv Rv1468c (gene ID: 886556) was then aligned with those acquired sequences in UBA family by using Jalview 2.10.2b2 (http://www.jalview.org/)^2^. For phylogenetic analysis, protein homologues of Mtb H37Rv Rv1468c in mycobacterial species were obtained by blasting of Mtb Rv1468c against UniProtKB_Bacteria database^3^ (E-threshold = 10). The phylogenetic tree was then constructed using Maximum Likelihood method by iTols online tool (http://itol.embl.de/)^4^.

**Supplementary References**

1 El-Gebali, S. *et al.* The Pfam protein families database in 2019. *Nucleic Acids Res* **47**, D427-D432, doi:10.1093/nar/gky995 (2019).

2 Waterhouse, A. M., Procter, J. B., Martin, D. M., Clamp, M. & Barton, G. J. Jalview Version 2–a multiple sequence alignment editor and analysis workbench. *Bioinformatics* **25**, 1189-1191, doi:10.1093/bioinformatics/btp033 (2009).

3 UniProt, C. UniProt: a worldwide hub of protein knowledge. *Nucleic Acids Res* **47**, D506-D515, doi:10.1093/nar/gky1049 (2019).

4 Letunic, I. & Bork, P. Interactive tree of life (iTOL) v3: an online tool for the display and annotation of phylogenetic and other trees. *Nucleic Acids Res* **44**, W242-245, doi:10.1093/nar/gkw290 (2016).
