# Supplementary material for: A Mycobacterium tuberculosis surface protein recruits ubiquitin to trigger host xenophagy
Source: Nat Commun. 2019 Apr 29;10:1973. doi: 10.1038/s41467-019-09955-8 (PMC6488588; doi:10.1038/s41467-019-09955-8)
Supplement: Supplementary file 3 — Description of Additional Supplementary Files [file 41467_2019_9955_MOESM3_ESM.docx]

**Description of Supplementary Files**

**File Name:** Supplementary Data 1

**Description:** List of variation sites in Mtb Rv1468c sequence based on 687 clinical isolates from GMTV database.

**File Name:** Supplementary Data 2

**Description:** Plasmids, bacterial strains and primers used in this study.
