# Supplementary material for: A Mycobacterium tuberculosis surface protein recruits ubiquitin to trigger host xenophagy
Source: Nat Commun. 2019 Apr 29;10:1973. doi: 10.1038/s41467-019-09955-8 (PMC6488588; doi:10.1038/s41467-019-09955-8)
Supplement: Supplementary file 6 — Reporting Summary [file 41467_2019_9955_MOESM6_ESM.pdf]

nature research

Corresponding author(s): Cui Hua Liu

Last updated by author(s): Mar 19, 2019

Reporting Summary

Nature Research wishes to improve the reproducibility of the work that we publish. This form provides structure for consistency and transparency in reporting. For further information on Nature Research policies, see Authors & Referees and the Editorial Policy Checklist.

Statistics

For all statistical analyses, confirm that the following items are present in the figure legend, table legend, main text, or Methods section.

n/a	Confirmed	
☐	☒	The exact sample size (*n*) for each experimental group/condition, given as a discrete number and unit of measurement
☐	☒	A statement on whether measurements were taken from distinct samples or whether the same sample was measured repeatedly
☐	☒	The statistical test(s) used AND whether they are one- or two-sided<br>*Only common tests should be described solely by name; describe more complex techniques in the Methods section.*
☒	☐	A description of all covariates tested
☐	☒	A description of any assumptions or corrections, such as tests of normality and adjustment for multiple comparisons
☐	☒	A full description of the statistical parameters including central tendency (e.g. means) or other basic estimates (e.g. regression coefficient) AND variation (e.g. standard deviation) or associated estimates of uncertainty (e.g. confidence intervals)
☐	☒	For null hypothesis testing, the test statistic (e.g. *F*, *t*, *r*) with confidence intervals, effect sizes, degrees of freedom and *P* value noted<br>*Give P values as exact values whenever suitable.*
☒	☐	For Bayesian analysis, information on the choice of priors and Markov chain Monte Carlo settings
☒	☐	For hierarchical and complex designs, identification of the appropriate level for tests and full reporting of outcomes
☒	☐	Estimates of effect sizes (e.g. Cohen's *d*, Pearson's *r*), indicating how they were calculated

Our web collection on statistics for biologists contains articles on many of the points above.

Software and code

Policy information about availability of computer code

Data collection	No software was used for data collection in this study.
Data analysis	GraphPad Prism 8.0, R 3.4.4, Jalview 2.10.2b2, and iTols online tool (http://itol.embl.de/).

For manuscripts utilizing custom algorithms or software that are central to the research but not yet described in published literature, software must be made available to editors/reviewers. We strongly encourage code deposition in a community repository (e.g. GitHub). See the Nature Research guidelines for submitting code & software for further information.

Data

Policy information about availability of data

All manuscripts must include a data availability statement. This statement should provide the following information, where applicable:

- Accession codes, unique identifiers, or web links for publicly available datasets
- A list of figures that have associated raw data
- A description of any restrictions on data availability

The source data underlying Figures 1c, 3c, e, 4a, b, e, f, 5b, d, 6b, 7a, d, e and 8a, and Supplementary Figs. 1c, 3e, f, 4e, f, 5a, d, e, 6b, c, 7b, d, 8b, 10c, e, g, 11d, and 12 are provided in Source Data. The original data of unprocessed blot and gel images are also available in Source Data. All other data that support the findings of this study are available from the corresponding author upon reasonable request.

Field-specific reporting

Please select the one below that is the best fit for your research. If you are not sure, read the appropriate sections before making your selection.

☒ Life sciences ☐ Behavioural & social sciences ☐ Ecological, evolutionary & environmental sciences

For a reference copy of the document with all sections, see nature.com/documents/nr-reporting-summary-flat.pdf

Life sciences study design

All studies must disclose on these points even when the disclosure is negative.

Sample size	According to our previous experiments of molecular biology and biochemistry, three independent experiments, each with 3-6 independent biological replicates could be sufficient to determine the differences between the tested groups. For immunofluorescence and electron microscopy analysis, a total of 100 or 200 bacterial cells of each group were analyzed, and these sizes were acceptable for observation and counting of each sample. For mice infection, we set five mice per group and performed three independent experiments, and this sample size was acceptable for the capacity of animal facility and sufficient for statistical analysis based on our experience of animal experiments.
Data exclusions	None.
Replication	Experiments were repeated at least three independent times. No experiment was found to be irreproducible.
Randomization	The experimental mice were allocated to each group based on their genotype. All experimental mice were age- and sex-matched in each experiment. No additional randomization or blinding was used to allocate experimental groups.
Blinding	There was no blinding being used.

Reporting for specific materials, systems and methods

We require information from authors about some types of materials, experimental systems and methods used in many studies. Here, indicate whether each material, system or method listed is relevant to your study. If you are not sure if a list item applies to your research, read the appropriate section before selecting a response.

Materials & experimental systems

n/a	Involved in the study	
☐	☒	Antibodies
☐	☒	Eukaryotic cell lines
☒	☐	Palaeontology
☐	☒	Animals and other organisms
☒	☐	Human research participants
☒	☐	Clinical data

Methods

n/a	Involved in the study	
☒	☐	ChIP-seq
☒	☐	Flow cytometry
☒	☐	MRI-based neuroimaging

Antibodies

Antibodies used	Rabbit anti-Mtb Rv1468c antibody was produced and purified by GenScript Biotechnology with the usage of reombinant His6-tagged Mtb Rv1468c protein as immunogen. The following commercially available antibodies were used in this study: anti-GFP (#ab1218; Abcam), anti-Flag (#F3165; Sigma-Aldrich), anti-HA (#3724; CST), mouse IgG1 (#ab18443; Abcam), anti-ubiquitin (#13-1600; Invitrogen), anti-ubiquitin specific for K63 (#ab179434; Abcam), anti-ubiquitin specific for K48 (#05-1307; Millipore), anti-LC3 (#L7543; Sigma-Aldrich), anti-LAMP1 (#ab24170; Abcam), anti-Galectin-3 (#ab2785; Abcam), anti-p62 (#NBP1-48320; Novus Biologicals), anti-GAPDH (#sc-25778; Santa Cruz), anti-Tubulin (#T5168, Sigma-Aldrich), anti-GST (#TA-03; ZSGB-BIO), anti-GroEL1 (#NBP2-32867; Novus Biologicals), anti-Ag85 (#ab36731, Abcam), and anti-Mtb (#ab905; Abacm).
Validation	Validation statements for antibodies used for immunoblot (GFP, Flag, HA, ubiquitin, LC3, GAPDH, Tubulin, GST, GroEL1 and Ag85) and immunofluorescence (HA, Ub, LC3, LAMP1, p62, K63 ubiquitin, K48 ubiquitin, Galectin-3 and Mtb) can be found on their corresponding manufacturer websites. Validation of Mtb Rv1468c antibody has been confirmed by using the sample of whole bacterial cell lysate of Mtb for immunoblot detection of a specific band at the predicted size with a negative control of the preimmune serum. The antibodies used for immunoelectron microscope (Ub, LC3, Rv1468c) were validated by using the ultrathin cryosection samples of mycobacteria or mycobacteria-infected BMDMs for detection of specific staining with a negative control of the isotype control antibody or the preimmune serum as described in the article.

Eukaryotic cell lines

Policy information about cell lines

Cell line source(s)	All cell lines used in this study were originally obtained from ATCC.
Authentication	No further authentication was made.
Mycoplasma contamination	All cell lines were tested negative for mycoplasma contamination.
Commonly misidentified lines (See ICLAC register)	No misidentified cell lines were used in this study.

Animals and other organisms

Policy information about studies involving animals; ARRIVE guidelines recommended for reporting animal research

Laboratory animals	Atg5 flox/flox mice (RBRC02975; RIKEN BRC) were provided by N. Mizushima (The University of Tokyo) with an Approval Form from the RIKEN BioResource Center; Lyz-Cre mice (stock number: 004781) were from the Jackson Laboratory. Experiments were performed with age- and sex-matched groups of 8-12 weeks old mice.
Wild animals	This study did not involve wild animals.
Field-collected samples	This study did not involve field-collected samples.
Ethics oversight	All mice were housed in a specific pathogen-free (SPF) facility on the basis of standard humane animal husbandry protocols, which were approved by the animal care and use committee of the Institute of Microbiology (Chinese Academy of Sciences). All animal studies were approved by the Biomedical Research Ethics Committee of Institute of Microbiology (Chinese Academy of Sciences).

Note that full information on the approval of the study protocol must also be provided in the manuscript.
